# Validity of visual assessment of aortic valve morphology in patients with aortic stenosis using two-dimensional echocardiography

**DOI:** 10.1007/s10554-020-02048-4

**Published:** 2020-10-11

**Authors:** Olena Nemchyna, Sajjad Soltani, Natalia Solowjowa, Felix Schoenrath, Yuriy Hrytsyna, Axel Unbehaun, Jörg Kempfert, Julia Stein, Christoph Knosalla, Andreas Hagendorff, Fabian Knebel, Volkmar Falk, Jan Knierim

**Affiliations:** 1 Department of Cardiothoracic and Vascular Surgery, German Heart Center Berlin, Augustenburger Platz 1, 13353 Berlin, Germany; 2 DZHK (German Centre for Cardiovascular Research), Partner Site Berlin, Hessische Straße 3- 4, 10115 Berlin, Germany; 3 Department of Cardiothoracic Surgery, Charité Universitätsmedizin Berlin, Corporate Member of Freie Universität Berlin, Humboldt-Universität zu Berlin, and Berlin Institute of Health, Charitéplatz 1, 10117 Berlin, Germany; 4 Department of Health Sciences and Technology, Translational Cardiovascular Technology, ETH Zurich, LFW C 13.2, Universitätstrasse 2, 8092 Zurich, Switzerland; 5 Department of Cardiology, Klinik und Poliklinik für Kardiologie, University of Leipzig, Liebigstraße 20, 04103 Leipzig, Germany; 6 Department of Cardiology and Angiology, Charité Universitätsmedizin Berlin, Corporate Member of Freie Universität Berlin, Humboldt-Universität zu Berlin, and Berlin Institute of Health, Campus Charité Mitte, Charitéplatz 1, 10117 Berlin, Germany

**Keywords:** Aortic valve stenosis, Two-dimensional echocardiography, Aortic valve calcium score, Visual assessment of aortic stenosis

## Abstract

The diagnostic value of a visual assessment of aortic valve (AV) morphology for grading aortic stenosis (AS) remains unclear. A visual score (VS) for assessing the AV was developed and its reliability with respect to Doppler measurements and the calcium score (ctCS) derived by multislice computed tomography was evaluated. 99 Patients with AS of various severity and 38 patients without AS were included in the analysis. Echocardiographic studies were evaluated using the new VS which includes echogenicity, thickening, localization of lesions and leaflet mobility, with a total score ranging from 0 to 11. The association of VS with ctCS and the severity of AS was analyzed. There was a significant correlation of VS with AV hemodynamic parameters and with ctCS. The cut-off value for the detection of AS of any grade was a VS of 6 (sensitivity 95%, specificity 85% for women; sensitivity 85%, specificity 88% for men). A VS of 9 for women and of 10 for men was able to predict severe AS with a high specificity (96% in women and 94% in men, AUC 0.8 and 0.86, respectively). The same cut-off values were identified for the detection of ctCS of ≥ 1600 AU and ≥ 3000 AU with a specificity of 77% and 82% (AUC 0.69 and 0.81, respectively). Assessment of aortic valve morphology can serve as an additional diagnostic tool for the detection of AS and an estimation of its severity.

**Electronic supplementary material:**

The online version of this article (10.1007/s10554-020-02048-4) contains supplementary material, which is available to authorized users.

## Background

Aortic stenosis (AS) is the most common valve disease, with a prevalence of 1.7% in people aged over 65 years and 3.4% in people aged over 75 years in North America and Europe [1]. Due to the ageing population it is expected that the prevalence of AS will continue to increase in the next decades. The pathophysiology of the disease is complex and in most cases includes fibro-calcific remodeling of the aortic valve, AV [2]. AV calcification is associated with AS severity and can be assessed by multislice computed tomography, MSCT [3], with a lower degree of AV calcification in women compared to men for the same severity of AS [4, 5].

According to the current guidelines, Doppler echocardiography is the gold standard for assessing AS severity [6]. However, around 30% of patients present with inconsistent echocardiographic findings [7, 8]. In these patients, grading of AS by Doppler measurements alone can be difficult and might require additional tests [8]. The assessment of AV calcification by MSCT is an important complementary approach [9]. It is already implemented in the guidelines for the assessment of patients with low-flow, low-gradient AS [10]. Furthermore, AV calcium load measured by MSCT has been shown to be of prognostic relevance in the natural course of AS [3–5] and in patients treated with percutaneous aortic valve replacement [11].

Valve morphology and degenerative changes of the valves can be assessed by two-dimensional echocardiography. It is also possible to estimate the degree of calcification by presence of increased echogenicity and thickening of the leaflets. Some studies demonstrated a correlation of the degree of cardiac calcium measured by MSCT with the echocardiographic calcium score [12]. A semi-quantitative grading of AV calcification has been proposed [10]. This approach was found to be of prognostic relevance for predicting the need for later AV replacement and mortality [13–16].

Although a visual assessment of valve morphology and leaflet movement is part of a comprehensive evaluation of AS, there are no studies linking morphological degenerative changes of the AV assessed by echocardiography to the calcium load measured by MSCT. There are also no data regarding a possible association of degenerative changes of the AV assessed by echocardiography with the severity of AS measured by Doppler gradients and the aortic valve area (AVA) determined by continuity equation.

*The aim of our study* was to investigate the reliability of a visual assessment of aortic valve morphology compared to the MSCT-derived calcium score and established Doppler parameters.

## Methods

### Study design and population

Clinical, echocardiographic and MSCT data of 153 adult patients who presented with normal AVs, AV sclerosis or degenerative aortic valve stenosis of various grades from July 2018 to October 2019 in a tertiary care cardiac surgery department were analyzed retrospectively. The majority of the patients were referred for AV replacement or for a second opinion regarding the AV. Patients without aortic valve pathology or with AV sclerosis were mostly referred for coronary artery bypass surgery.

Patients in whom echocardiography with an assessment of the AV was indicated were considered eligible for the analysis. No additional tests, in particular no MSCT, were performed for the study. Exclusion criteria were: congenital heart disease, previous aortic valve surgery, rheumatic heart disease, endocarditis, bicuspid aortic valve. All patients underwent routine transthoracic echocardiography. 16 Patients had to be excluded because of insufficient image quality for a visual assessment of the AV. In total, 137 patients were included in the final analysis.

The study was approved by the Ethics Committee at Charité University (Ref. No. EA2/058/19). Research was performed in accordance with the Declaration of Helsinki.

### Transthoracic echocardiography

Echocardiographic studies were performed using Vivid S70 (GE Vingmed Ultrasound, Horton, Norway; transducer M5Sc-D, 1.4–4.6 MHz), Vivid-E9 (GE Vingmed Ultrasound, Horton, Norway; transducer M5S-D, 1.4–4.6 MHz) and Philips EPIQ 7G (Philips Medical Systems, Andover, MA, USA; transducer X5-1, 1–5 MHz) ultrasound machines and stored in the Institutional Data Repository. All echocardiographic studies were conducted by experienced clinicians. Image analysis and all measurements were carried out according to the current guidelines [11, 17]. At the time of recording, maximum efforts were made to obtain optimal aortic valve images using zoom mode in most cases and manual adjustments of the gain and dynamic range according to the recommendations [18]. Two-dimensional images in the parasternal long-axis view, parasternal short-axis view, as well as three- and five-chamber apical views of the left ventricle (LV) focused on the AV were recorded in standard Grey scale using harmonic imaging mode and stored for most studies.

### Classification and inclusion of patients

As recommended by current guidelines, the severity of AS was based on the peak jet velocity across the aortic valve, the mean transvalvular pressure gradient, and the effective aortic valve area by continuity equation [6, 19]. Thus, severe AS was assumed only in cases with AVA < 1.0 cm^2^ and peak jet velocity ≥ 4 m/s and/or a mean gradient ≥ 40 mmHg. Patients with visual degenerative changes of the aortic valve without signs of obstruction of left ventricular outflow tract were classified as having aortic sclerosis.

In the case of incongruent data in AVA, peak jet velocity and mean gradient, such as patients with low-flow, low-gradient AS, the results were labelled as inconsistent grading.

**Fig. 1:**
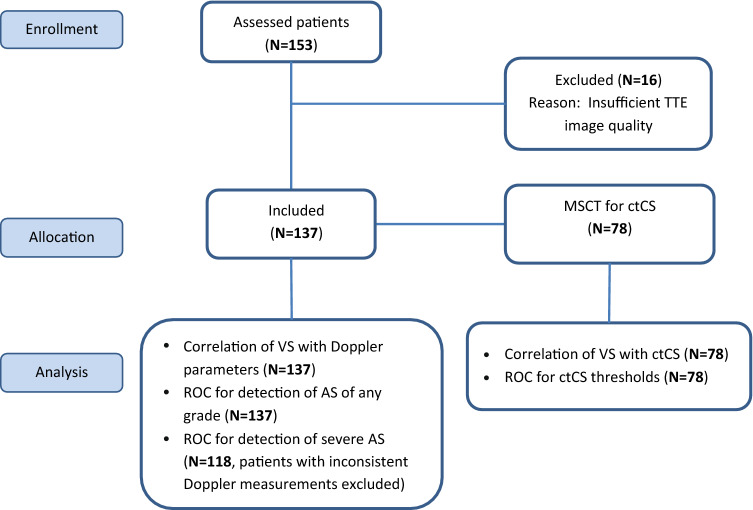
Flowchart of patient dispositions and analysis performed. *TTE* transthoracic echocardiography, *MSCT* multislice computed tomography, *ctCS* calcium score derived by computed tomography, *VS* visual score, *AS* aortic stenosis

Data of patients with an inconsistent grading of AS severity were not included in the analysis of diagnostic accuracy for detecting severe AS using the visual score, but they were included in the analysis of the association of the visual score with the AV calcium score obtained by MSCT. For details see Fig. 1.

### Visual assessment of aortic valve

Echocardiographic studies were anonymized; loops and images obtained by Doppler technique and all measurements based on Doppler were deleted. Studies were labelled with the unique study number and uploaded into a digital database (IntelliSpace Cardiovascular 4.1, Koninklijke Philips N.V., Netherlands). Anonymized echocardiographic examinations were re-assessed retrospectively by one investigator. At this time, visual grading of AV morphology and scoring of degenerative changes was performed using two-dimensional images only. Degenerative changes of the AV were evaluated using a visual score comprising four characteristics: echogenicity, thickening, localization of valve lesions, and mobility of AV leaflets (Table 1). The minimum possible score was 0 and the maximum was 11.

**Table 1:** Grading system of aortic valve morphological changes

Parameter	Criteria of assessment	Score
Calcification	– No changes	0
	– Mildly increased echogenicity	1
	– Moderately increased echogenicity	2
	– Severely increased echogenicity with signs of acoustic shadowing	3
Thickening	– No changes	0
	– Mildly thickened leaflets	1
	– Moderately thickened leaflets	2
	– Severely thickened leaflets	3
Localization of lesions	– No lesion	0
	– Lesion of one leaflet in long-axis view and/or in short-axis view	1
	– Lesion of both leaflets in long-axis view and/or of two or three leaflets in short-axis view	2
Leaflet mobility	– Normal opening of leaflets	0
	– Mildly restricted opening/mobility	1
	– Moderately restricted opening/mobility	2
	– Severely restricted opening/mobility	3
Maximum possible score		11

To assess the inter-observer variability in the VS and the visual assessment of AS, 40 randomly selected studies were graded by an independent experienced investigator using the same visual grading approaches. For intra-observer variability, these 40 studies were graded by the same observer ≥ 1 month after the initial grading.

### Multislice computed tomography

Whenever MSCT was indicated in the clinical setting (mainly for planning of a transcatheter aortic valve replacement procedure), the data were used for our analysis provided that less than 3 months had passed between the echocardiography and MSCT. The non-contrast ECG-gated cardiac scanning was performed using a second-generation dual-source scanner (SOMATOM Definition Flash, Siemens AG, Erlangen, Germany) with a reference tube current of 80 mA (using CARE Dose 4D) and a tube voltage of 120 kV. Images were analyzed using validated software (syngo.via CT CaScoring, Siemens AG, Erlangen, Germany) with the Agatston method to quantify the degree of AV calcium [20] on contiguous 3 mm multiplane slices under exclusion of calcium originating from the mitral valve annulus, the ascending aorta, and the coronary arteries. The total calcium score was calculated semi-automatically with a threshold of 130 Hounsfield units. This method is validated in our center and shows excellent results regarding the ability to detect severe aortic stenosis with the cut-offs for severe AS close to those published by other groups [5, 7] (Supplementary Fig. 1). The cardiologist performing the AV calcium scoring was blinded to the results of the echocardiographic examinations.

### Statistical analysis

All continuous variables are presented as mean (± SD) or median (with interquartile intervals) where appropriate. Categorical variables are presented as numbers with percentages. Differences between continuous variables were estimated with the independent samples t-test, differences between categorical variables were evaluated by χ^2^ test and Fisher’s exact test. The Pearson correlation coefficient was used to estimate the correlation between continuous variables; Spearman’s correlation coefficient was applied for categorical variables. The diagnostic value of VS for detecting conditions of interest was analyzed using receiver-operating curves (ROC). For the detection of AS of any grade data on whole population was used for ROC analysis, for the detection of severe AS only patients with consistent measurements for AS severity were included in ROC analysis (Fig. 1). Inter-observer and intra-observer agreement was assessed using the interclass correlation coefficient (ICC); results for variability in measurements were presented as Bland–Altman plots. Statistical significance was defined by p < 0.05. The data were analyzed using SPSS 24 (SPSS, Chicago, IL, USA) and R, version 3.5.2.

## Results

### Baseline patient characteristics

137 Patients (mean age 74.7 ± 9.4 years, 36.5% women) were included in this study. Clinical, echocardiographic and MSCT patient characteristics are summarized in Table 2. Patients with AS were older and exhibited a similar prevalence of comorbidities compared to those without AS. Men had a higher prevalence of coronary artery disease compared to women, as well as greater mean LV volumes and LV mass and a lower mean stroke volume index and LV ejection fraction. Hemodynamic parameters of AS were similar between female and male patients. In patients with severe AS, the average mean gradient was 45.8 ± 7.1 mmHg, the average peak aortic jet velocity was 4.3 ± 0.3 m/s and the average AVA was 0.67 ± 0.2 cm^2^, without a significant difference in these parameters between female and male patients (Supplementary Table 1).

**Table 2:** Patient characteristics

Parameter	All patients	Groups by aortic stenosis (AS)			Groups by sex		
		Patients with AS	Patients without AS	p-value	Women	Men	p-value
Number of patients	137	99 (72.3)	38 (27.2)	–	50 (36.5)	87 (63.5)	–
Age (years) Range	74.7 ± 9.4 35–90	77.1 ± 7.3 57–90	68.5 ± 11.3 35–88	< 0.0001	75.7 ± 8.5 55–90	74.1 ± 9.8 35–90	0.322
Women	50 (36.5)	37 (37.4)	13 (34.2)	0.844	–	–	–
Body mass index (kg/m^2)^	26 ± 4.9	26.6 ± 4.5	24.5 ± 5.3	0.023	25.9 ± 5.2	26.1 ± 4.6	0.78
Body surface area (m^2)^	1.9 ± 0.2	1.89 ± 0.2	1.89 ± 0.2	0.95	1.73 ± 0.2	1.98 ± 0.1	< 0.0001
Coronary artery disease	83 (60.6)	61 (61.6)	22 (57.9)	0.7	24 (48)	59 (67.8)	0.029
Diabetes mellitus	34 (24.8)	28 (28.3)	6 (15.8)	0.2	9 (18)	25 (28.7)	0.22
Arterial hypertension	108 (78.8)	82 (82.8)	26 (68.4)	0.1	37 (74)	71 (81.6)	0.385
Atrial fibrillation at the time of TTE	11 (8)	9 (9.1)	2 (5.3)	0.6	3 (6)	8 (9.2)	0.593
Echocardiography							
HR (bpm)	71.5 ± 14.7	71.6 ± 13.9	71.3 ± 16.6	0.93	72.2 ± 14	71.1 ± 15	0.67
LV EDDI (mm/m^2)^	25.3 ± 4.5	25.2 ± 4.3	25.5 ± 5.1	0.77	25 ± 4.6	25.5 ± 4.5	0.56
LV EDVI (mL/m^2)^	60.9 ± 24	59.9 ± 22.6	56 ± 14.9	0.45	49.8 ± 15.5	67.2 ± 25.7	< 0.0001
LV ESVI (mL/m^2)^	28.6 ± 20.4	27.9 ± 18.1	30.5 ± 25.6	0.51	20.9 ± 13.4	32.9 ± 22.4	< 0.0001
LV EF (%)	56.8 ± 13.7	57.1 ± 13.3	56 ± 14.9	0.67	60.1 ± 12.6	55 ± 14	0.039
LV SVI (Doppler) (mL/m^2)^	36.2 ± 9.2	36.8 ± 9.8	34.6 ± 7.2	0.21	37.5 ± 9.3	35.3 ± 9	< 0.0001
LV mass index (g/m^2)^	131 ± 40	136 ± 40	119 ± 39	0.02	118 ± 35	139 ± 42	0.004
Aortic valve parameters							
Peak velocity (m/s)	3 ± 1.1	3.6 ± 0.7	1.6 ± 0.5	< 0.0001	3.1 ± 1.1	2.9 ± 1.1	0.38
Mean gradient (mmHg)	24.5 ± 16.7	31.8 ± 13.8	5.5 ± 3.4	< 0.0001	25.6 ± 17.4	23.9 ± 16.4	0.58
AVA (cm^2)^	1.25 ± 0.7	0.9 ± 0.3	2.2 ± 0.7	< 0.0001	1.15 ± 0.7	1.3 ± 0.7	0.23
Aortic valve abnormality							0.79
Normal AV	20 (14.6)	–	20 (52.6)	–	6 (12)	14 (16.1)	
Aortic sclerosis	18 (13.1)	–	18 (47.4)	–	7 (14)	11 (12.6)	
Mild AS	13 (9.5)	13 (13.1)	–	–	5 (10)	8 (9.2)	
Moderate AS	26 (19)	26 (26.3)	–	–	7 (14)	19 (21.8)	
Severe AS	41 (29.9)	41 (41.4)	–	–	18 (36)	23 (26.4)	
AS of inconsistent grading	19 (13.9)	19 (19.2)	–	–	7 (14)	12 (13.8)	
MSCT for AV calcium score							
Number of pts with MSCT data, n	78	74	4	–	32	46	–
Aortic valve abnormality							0.62
Aortic sclerosis	4 (5.1)	–	–	–	2 (6.3)	2 (4.3)	
Mild AS	3 (3.8)	–	–	–	2 (6.3)	1 (2.2)	
Moderate AS	12 (15.4)	–	–	–	3 (9.4)	9 (19.6)	
Severe AS	40 (51.3)	–	–	–	18 (56.3)	22 (47.8)	
AS of inconsistent grading	19 (24.4)	–	–	–	7 (21.9)	12 (26.1)	
Mean calcium score (AU)	2467 ± 1809	2571 ± 1797	539 ± 353	0.028	1753 ± 1156	2963 ± 2016	0.001
Calcium score distribution				0.02			0.033
< 800	9 (11.5)	6 (8.1)	3 (75)		6 (18.8)	3 (6.5)	
800–1199	8 (10.3)	7 (9.5)	1 (25)		4 (12.5)	4 (8.7)	
1200–1599	14 (17.9)	14 (18.9)	0		9 (28)	5 (11)	
1600–1999	11 (14.1)	11 (14.9)	0		3 (9.4)	8 (17.4)	
2000–2999	13 (16.7)	13 (17.6)	0		6 (18.8)	7 (15.2)	
≥ 3000	23 (29.5)	23 (31.1)	0		4 (12.5)	19 (41.3)	

Values are mean (± SD), median (interquartile range), or n (%)

*HR* heart rate, *EDDI* end-diastolic diameter index, *EDVI* end-diastolic volume index, *ESVI* end-systolic volume index, *EF* ejection fraction, *SVI* stroke volume index, *TTE* transthoracic echocardiography

### Calcium scoring by MSCT

Calcium scoring by MSCT was performed in 78 patients (32 female and 46 male). Most patients who underwent MSCT had moderate or severe AS. There were four patients without AS, but with the aortic valve sclerosis. The mean aortic valve calcium score by MSCT (ctCS) of all patients was 2467 ± 1809 Agatston units (AU) with a higher mean score in men than in women (2963 ± 2016 AU vs. 1753 ± 1156 AU, p = 0.001). See Table 2 for details. The mean ctCS in male patients with severe AS was 4188 ± 2084 AU and in female patients 2301 ± 1214 AU (p = 0.002).

### Visual scoring: intra- and inter-observer validation

**Fig. 2:**
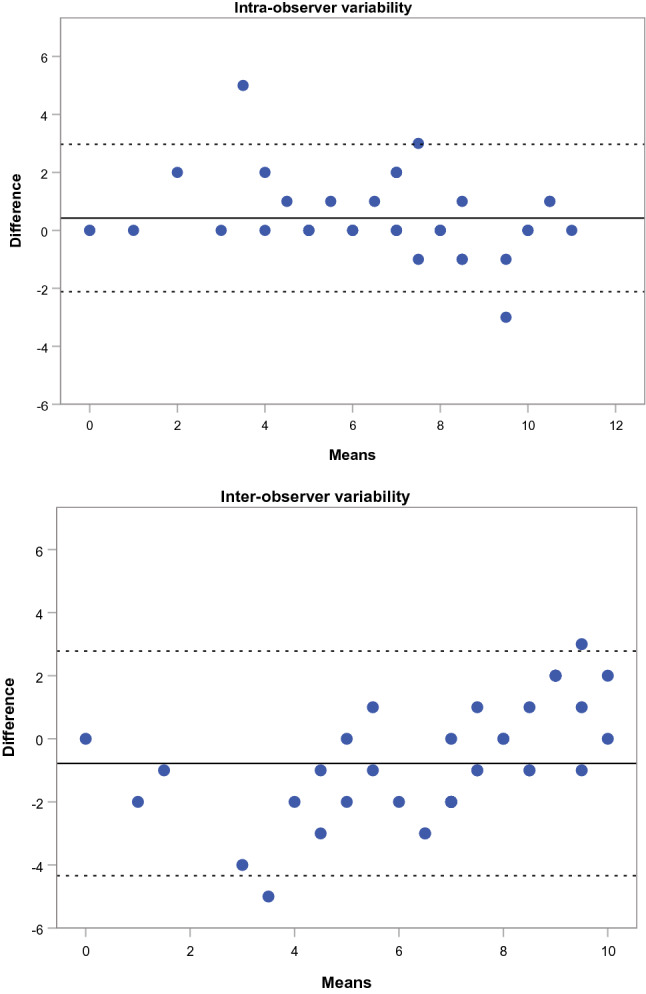
Bland–Altman plots of intra- and inter-observer agreement in AV visual score (for intra-observer plot 95% limits of agreement − 2.16, 2.97, mean difference 0.43, for inter-observer plot 95% limits of agreement − 4.34, 2.79, mean difference − 0.78)

There was good intra- and inter-observer agreement in the assessment of the AV using the visual score as demonstrated by the ICC of 0.95 (95% CI 0.90–0.97, p < 0.0001) and 0.86 (95% CI 0.74–0.93, p < 0.0001), respectively. The absolute difference in the VS between observers was ≤ 1 in 45% of cases, ≤ 2 in 85% of cases, and > 2 in 15% of cases. The absolute difference in VS measured by the same observer was ≤ 1 in 80% of cases, ≤ 2 in 92.5% of cases, and > 2 in 7.5% of cases. No significant difference in the inter-observer grading for single parameters of VS was observed (Supplementary Table 2). For the intra- and inter-observer agreement in VS see Fig. 2 for the Bland–Altman plot.

### Visual scoring and echocardiographic measures

**Fig. 3:**
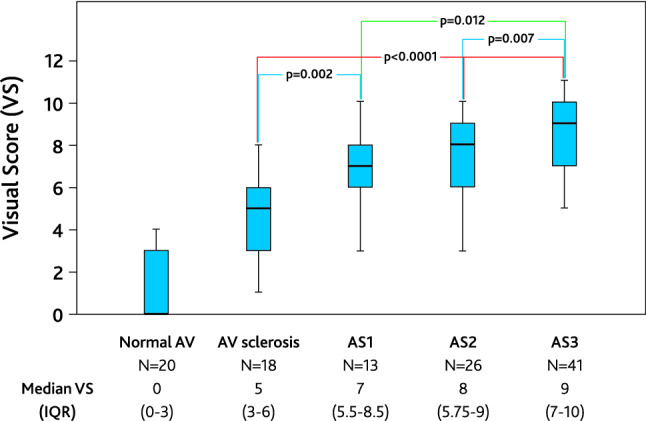
Visual score (VS) in groups by AV abnormality. Boxes indicate the 25th to 75th percentiles, the line within the boxes indicate median value, the vertical line indicates 95% range for VS. Differences between groups are shown when significant. p < 0.0001 between patients with normal AV and each other group. p < 0.0001 for Kruskal–Wallis test

The median visual score (VS) was significantly higher in patients with AS than in patients without AS, irrespective of whether the criterion “mobility” was included in the score (Table 3), and the VS increased with the grade of aortic stenosis (Fig. 3).

**Table 3:** Results of visual assessment of the aortic valve

Parameter	All patients	Patients with AS	Patients without AS	p-value	Women	Men	p-value
Number of patients	137	99 (72.3)	38 (27.2)	–	50 (36.5)	87 (63.5)	–
Image quality				0.003			0.62
Good	21 (15.3)	9 (9)	12 (31.6)		9 (18)	12 (13.8)	
Moderate	116 (84.7)	90 (91)	28 (68.4)		41 (82)	75 (86.2)	
Median visual score							
Visual score	7 (5–9)	8 (6–10)	3 (0–5)	< 0.0001	7 (5–8.25)	7 (5–9)	0.83
Visual score (excluding mobility)	5 (4–7)	6 (5–7)	3 (0–4)	< 0.0001	5 (4–6)	5 (4–7)	0.8

Values are n (%) or median (interquartile range)

There was a good correlation of VS with peak aortic jet velocity (r = 0.64, p < 0.0001), mean transvalvular aortic gradient (r = 0.65, p < 0.0001), and aortic valve area calculated by continuity equation (r = − 0.69, p < 0.0001). The correlation between VS and the hemodynamic parameters measured by echocardiography was noticeably higher compared to the correlation between ctCS and the same parameters (Table 4).

**Table 4:** Correlation data

Parameter	Visual score		Calcium score by MSCT	
	Spearman’s correlation coefficient	p-value	Pearson correlation coefficient	p-value
All patients, n = 137				
Calcium score by MSCT	0.496	< 0.0001	–	–
Peak aortic jet velocity	0.643	< 0.0001	0.544	< 0.0001
Mean gradient	0.653	< 0.0001	0.581	< 0.0001
AVA	− 0.687	< 0.0001	− 0.274	0.015
Women, n = 50				
Calcium score by MSCT	0.582	0.0005	–	–
Peak aortic jet velocity	0.58	< 0.0001	0.548	0.001
Mean gradient	0.614	< 0.0001	0.582	0.0005
AVA	− 0.584	< 0.0001	− 0.45	0.01
Men, n = 87				
Calcium score by MSCT	0.476	0.001	–	–
Peak aortic jet velocity	0.676	< 0.0001	0.637	< 0.0001
Mean gradient	0.678	< 0.0001	0.671	< 0.0001
AVA	− 0.751	< 0.0001	− 0.274	0.065

**Fig. 4:**
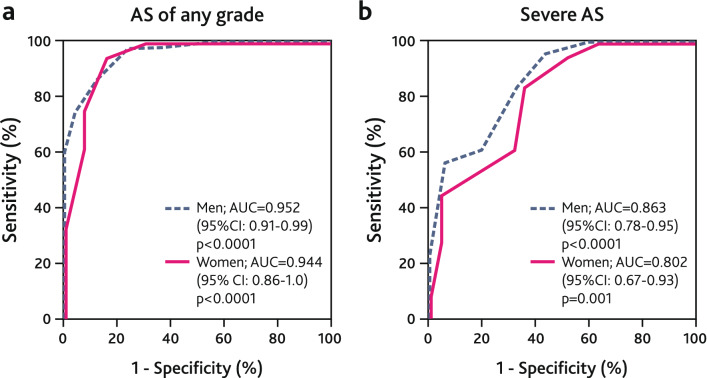
ROC curves for the detection of AS of any grade (**a**) and severe AS (**b**) by visual score in women and men

A VS of 6 was identified as the optimal cut-off for detecting AS of any grade in women and men, with a sensitivity of 95% and a specificity of 85% in women and a sensitivity of 85% and a specificity of 88% in men. On the other hand, a VS of 9 had a sensitivity of 44% and a specificity of 96% in women to predict severe AS. In men a VS of 10 had a sensitivity of 56% and a specificity of 94% to predict severe aortic stenosis (Table 5; Fig. 4).

**Table 5:** Diagnostic accuracy of visual score in detecting AS

	VS cut-off	Sensitivity (%)	Specificity (%)	PPV (%)	NPV (%)	AUC (95% CI)	p-value
Women							
AS of any grade	6	95	85	95	85	0.944 (0.86–1.0)	< 0.0001
Severe AS	9	44	96	89	71	0.802 (0.67–0.93)	0.001
Men							
AS of any grade	6	85	88	95	71	0.952 (0.91–0.99)	< 0.0001
Severe AS	10	56	94	81	83	0.863 (0.78–0.95)	< 0.0001

*PPV* positive predictive value, *NPV* negative predictive value

### Visual scoring and calcium scoring by MSCT

There was a positive correlation between VS and calcium score measured by MSCT (r = 0.496, p < 0.0001) (Table 4).

The ROC analysis identified a VS of 9 as optimal for detecting a ctCS of ≥ 1600 AU with a sensitivity of 55% and a specificity of 77% (AUC 0.69, 95% CI 0.57–0.81, p = 0.005). A VS of 10 was able to detect a ctCS of ≥ 3000 AU with a sensitivity of 70% and a specificity of 82% (AUC 0.81, 95% CI 0.72–0.9, p < 0.0001) (Supplementary Fig. 2).

### Visual score of selected parameters and parameter combinations

The correlation of the different parameters of the VS with MSCT and echo parameters was analyzed. Combinations of parameters performed better than single parameters. Calcification and thickening showed a stronger correlation than localization and movement (Supplementary Table 3). An additional analysis excluding the mobility pattern was performed for the VS. This VS also demonstrated good inter-observer agreement, with an ICC of 0.85 (95% CI 0.72–0.92, p < 0.0001) (Supplementary Fig. 3) and a good correlation with hemodynamic parameters of AS severity and with ctCS (Supplementary Table 4). A ROC analysis was performed. The cut-off value for VS without the mobility pattern for detecting AS of any grade in women and men was 5. The cut-off value for detecting severe AS was 7 with a high specificity in women (96%) and men (90%). VS without mobility was able to detect ctCS of ≥ 1600 AU and ctCS of ≥ 3000 AU with a specificity of 81% and 93%, respectively (for details see Supplementary Table 5).

## Discussion

Some studies have shown that patients with severe AV calcification (as assessed by echocardiography) have worse outcomes irrespective of the severity of valvular dysfunction [14, 16]. However, calcification assessed by MSCT and echocardiographic characteristics of AV has not been previously compared.

To our knowledge this is the first study comparing the visual assessment of morphological changes of the AV with the Doppler measurements and the degree of calcification obtained by MSCT.

We established an easily applicable semi-quantitative visual score (VS) for grading degenerative changes of the AV (Table 1). Our grading system includes a range of patterns: thickening of leaflets, echogenicity, localization of lesions, and mobility of leaflets. In contrast to the generally accepted visual assessment of AV calcification, with grading into the categories mild, moderate and severe [10], the proposed score offers a wider range with a minimum of 0 and a maximum of 11 points. This approach performed better than any one single parameter and provides complementary information as degenerative aortic valve disease is not characterized by calcification alone. The established score showed good intra- and inter-observer validity also when the observer was blinded for color Doppler images and Doppler measurements (Fig. 2).

VS showed a good correlation with peak aortic jet velocity, mean transvalvular aortic gradient, and AVA calculated by continuity equation. The correlation was even stronger than the correlation of ctCS with the same parameters. VS also demonstrated a significant correlation with ctCS (Table 4).

A VS of < 6 was able to exclude any kind of aortic stenosis with a high sensitivity and specificity (Table 5). Echocardiography is good tool for detecting movement, calcification and thickening, but fusion of the valves can be overseen. This may explain the limited sensitivity of the VS for detecting severe AS. However, severe AS was highly likely for a VS equal to or higher than 9 (in women) and 10 (in men) (Table 5).

**Fig. 5:**
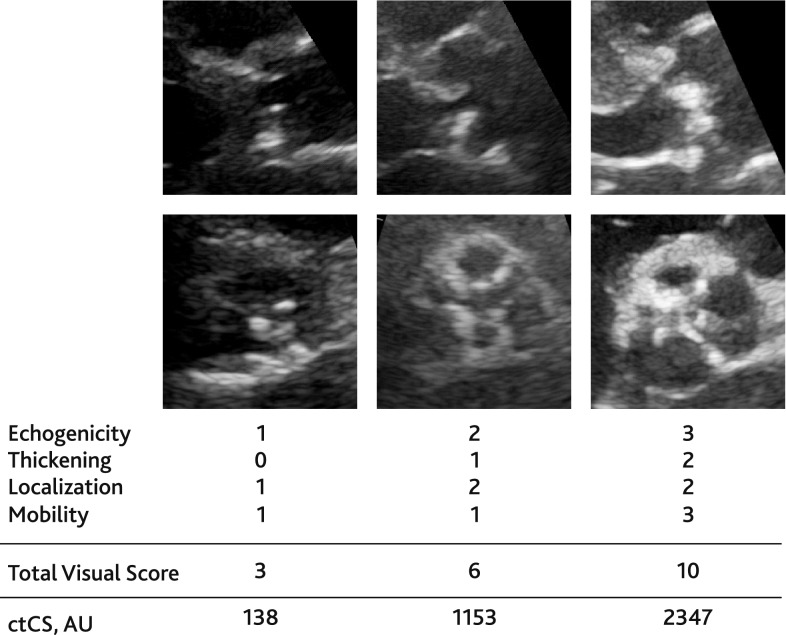
Examples on visual scoring in patients with various aortic valve abnormality. On the left—patient with aortic sclerosis, in the middle—patient with mild aortic stenosis, on the right—patient with severe aortic stenosis. See Online Resources for complimentary video-loops (ESM_1 and ESM_2 for visual score of 3, ESM_3 and ESM_4 for visual score of 6, ESM_5 and ESM_6 for visual score of 10)

The simple visual assessment of the aortic valve was specific for the detection of MSCT thresholds of severe AV calcification in women (≥ 1600 AU) and men (≥ 3000 AU) (Supplementary Fig. 2) as suggested by current guidelines [10]. Figure 5 demonstrates visual grading of AV with various abnormalities.

Although the MSCT-derived calcium score is widely accepted, easily obtained and quantified, it is associated with additional risks for the patient and is not always available. Experienced physicians and sonographers intuitively consider the visual aspect of the AV when performing echocardiography and interpreting Doppler measurements. Establishing a reproducible and more objective visual grading system may help to standardize this common practice and may offer guidance in unclear and inconsistent cases. The current results show that visual scoring can add important information to Doppler measurements. VS can exclude AS and confirm severe AS. This approach might be especially important in a setting where additional tests like MSCT are not always available due to limited resources.

Another possible scenario is the use of 2D echocardiography as a screening tool and point-of-care approach when ultrasound examinations could be done by nurses or general practitioners without expertise in echocardiography, also when insufficient or no Doppler images are acquired.

**Fig. 6:**
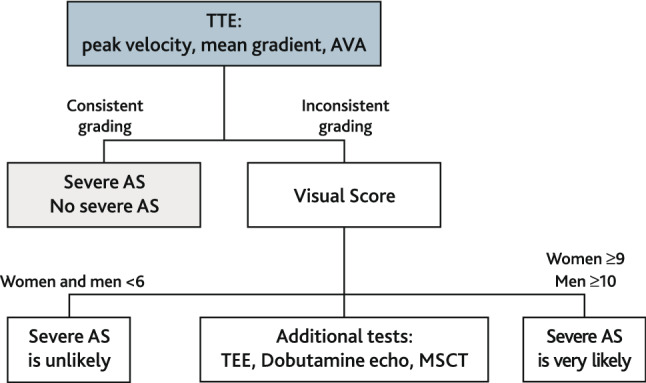
Clinical application of the visual score for the assessment of aortic stenosis. *TTE* transthoracic echocardiography, *TEE* transesophageal echocardiography

We also propose including the VS in the assessment of AS severity in patients with inconsistent AS grading, as demonstrated in the flow chart (Fig. 6). This approach has the potential to reduce the need for additional tests, like transesophageal echocardiography, MSCT or stress echocardiography in cases where the visual assessment clearly suggests severe AS.

A potential field of application for the VS is low-gradient AS. This particular group of patients with inconsistent grading is of special interest as the proposed diagnostic workup is time-consuming and may cause a delay in diagnosis and treatment [10].

Including the parameter “mobility” in a grading system to evaluate low-gradient stenosis could be misleading. The additional analysis of VS without the “mobility” pattern yielded similar results in correlation with hemodynamic parameters and ctCS, as well as a comparable diagnostic ability to detect severe AS and to determine the ctCS thresholds. This reliability of the VS without the “mobility” pattern could make the VS important for assessing low-flow, low-gradient AS, although this has to be investigated in a larger, prospective study.

## Limitations

An important limitation of the VS is its subjectivity. Although we found good inter-observer agreement there will be discrepancies in the visual estimation of thickening/calcification and all other parameters applied. Nevertheless, a visual assessment of morphology and movement is an important part of a comprehensive evaluation of each individual valve and needs to be included in the final echo interpretation. The VS makes this subjective impression more comparable and underlines its importance in the estimation of AS severity.

Another limitation is that echocardiographic studies were performed using different ultrasound machines; moreover, sonographers might have used different ultrasound settings for image optimization. This, however, represents the real-life situation. Furthermore, the investigator who performed the visual scoring was able to compare the echogenicity of other heart structures with the echogenicity of the AV, which may have influenced the visual assessment.

Furthermore, MSCT was performed only when indicated in the clinical setting. Therefore, MSCT data were mostly available in patients with severe and not in patients with mild or moderate AV calcification. This may have influenced the correlation of VS and Doppler parameters with ctCS. It may also have reduced the sensitivity of VS to identify patients with severe calcification measured by MSCT.

The study was conducted in a single center; therefore, it is possible that visual scores obtained in other centers might differ systematically from our results. However, we found a good agreement between two observers who did not perform the echocardiography and were blinded for Doppler measurements and MSCT results.

## Conclusions

Assessing AV morphology using a simple semi-quantitative visual score (VS) is feasible. The VS demonstrated a good correlation with Doppler measurements and the calcium score obtained by MSCT. It allowed for the exclusion of AS of any grade and the detection of severe AS. Therefore, a visual assessment of the aortic valve during echocardiography might be used in certain clinical settings as part of an integrated approach for evaluating the aortic valve.

## Electronic supplementary material

Below is the link to the electronic supplementary material.

(AVI 12697 kb)

(AVI 14051 kb)

(AVI 16973 kb)

(AVI 16621 kb)

(AVI 16777 kb)

(AVI 17760 kb)

(DOCX 144 kb)

## Data Availability

Available.

## Abbreviations

AS: Aortic stenosis
AV: Aortic valve
AVA: Aortic valve area
ctCS: Calcium score derived by MSCT
LV: Left ventricle
MSCT: Multislice computed tomography
ROC: Receiver-operating curve
VS: Visual score
