# Supplementary material for: Validity of visual assessment of aortic valve morphology in patients with aortic stenosis using two-dimensional echocardiography
Source: Int J Cardiovasc Imaging. 2020 Oct 11;37(3):813–23. doi: 10.1007/s10554-020-02048-4 (PMC7969560; doi:10.1007/s10554-020-02048-4)
Supplement: Supplementary file 7 — (DOCX 144 kb) [file 10554_2020_2048_MOESM7_ESM.docx]

**Suppl. Table 1. Hemodynamic parameters in patients with severe AS.**

| **Parameter** | **All patients**  **n=41** | **Women**  **n=18** | **Men**  **n=23** | **p-value** |
| --- | --- | --- | --- | --- |
| Peak aortic jet velocity, m/s | 4.3±0.3 | 4.3±0.4 | 4.3±0.3 | 0.85 |
| Mean gradient, mmHg | 45.8±7.1 | 45.3±8.9 | 46.2±5.6 | 0.67 |
| AVA, cm^2^ | 0.67±0.2 | 0.61±0.2 | 0.73±0.2 | 0.06 |
| SVI, mL/m^2^ | 38.2±10.1 | 36.4±10.4 | 39.7±9.8 | 0.3 |

**Suppl. Table 2. Inter-observer variability for grading of single parameters of VS.**

|  | Mean difference ±SD | p-value | Interclass correlation coefficient  (95% CI) | p-value |
| --- | --- | --- | --- | --- |
| Calcification | 0.2±0.7 | 0.07 | 0.79 (0.59-0.89) | <0.0001 |
| Thickening | 0.17±0.6 | 0.09 | 0.81 (0.64-0.9) | <0.0001 |
| Localization | 0.15±0.5 | 0.06 | 0.76 (0.54-0.87) | <0.0001 |
| Mobility | 0.25±0.8 | 0.05 | 0.75 (0.53-0.87) | <0.0001 |

**Suppl. Table 3. Correlation of VS and its separate parameters with Doppler parameters and with ctCS.**

| **Parameter** | **Calcification** | **Thickening** | **Localization** | **Mobility** | **VS** | **VS excluding mobility** | **VS excluding localization** | **VS excluding mobility and localization** |
| --- | --- | --- | --- | --- | --- | --- | --- | --- |
| Calcium score by MSCT | 0.42* | 0.41* | 0.21 | 0.4* | 0.5* | 0.5* | 0.48* | 0.48* |
| Peak aortic jet velocity | 0.61* | 0.63* | 0.51* | 0.57* | 0.64* | 0.65* | 0.64* | 0.65* |
| Mean gradient | 0.62* | 0.64* | 0.51* | 0.58* | 0.65* | 0.66* | 0.65* | 0.66* |
| AVA | -0.65* | -0.66* | -0.5* | -0.63* | -0.69* | -0.69* | -0.69* | -0.69* |

- **p<0.0001**

**Suppl. Table 4. Correlation of visual score excluding mobility pattern with AV calcium score hemodynamic parameters.**

| **Parameter** | **Correlation coefficient** | **p-value** |
| --- | --- | --- |
| **All patients** | | |
| Calcium score by MSCT | 0.496 | <0.0001 |
| Peak aortic jet velocity | 0.663 | <0.0001 |
| Mean gradient | 0.65 | <0.0001 |
| AVA | -0.685 | <0.0001 |
| **Women** | | |
| Calcium score by MSCT | 0.579 | 0.001 |
| Peak aortic jet velocity | 0.61 | <0.0001 |
| Mean gradient | 0.579 | <0.0001 |
| AVA | -0.578 | <0.0001 |
| **Men** | | |
| Calcium score by MSCT | 0.46 | 0.001 |
| Peak aortic jet velocity | 0.687 | <0.0001 |
| Mean gradient | 0.684 | <0.0001 |
| AVA | -0.748 | <0.0001 |

**Suppl. Table 5. Diagnostic accuracy of VS without mobility pattern in detecting AS and ctCS thresholds.**

|  | **VS cut-off** | **Sensitivity, %** | **Specificity, %** | **AUC (95%CI)** | **p-value** |
| --- | --- | --- | --- | --- | --- |
| Women | | | | | |
| AS of any grade | 5 | 89 | 92 | 0.95 (0.86-1.0) | <0.0001 |
| Severe AS | 7 | 39 | 96 | 0.79 (0.66-0.92) | 0.001 |
| Men | | | | | |
| AS of any grade | 5 | 84 | 88 | 0.94 (0.89-0.99) | <0.0001 |
| Severe AS | 7 | 65 | 90 | 0.87 (0.78-0.95) | <0.0001 |
| Women and men | | | | | |
| ctCS ≥ 1600 AU | 7 | 53 | 81 | 0.68 (0.56-0.8) | 0.007 |
| ctCS ≥ 3000 AU | 8 | 30 | 93 | 0.81 (0.71-0.91) | 0.001 |

**Women Men**


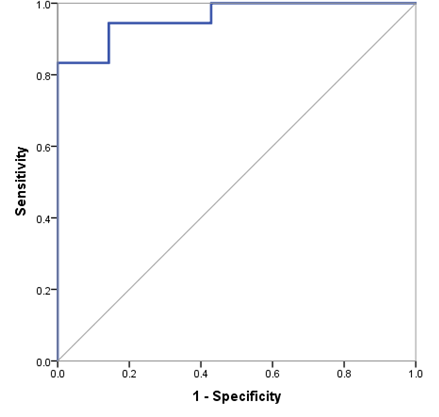

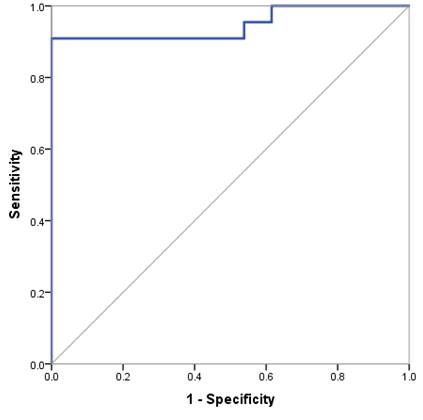


|  | **ctCS cutoff** | **Sensitivity,**  **%** | **Specificity,**  **%** | **PPV,**  **%** | **NPV,**  **%** | **AUC, %**  **(95% CI)** | **p-value** |
| --- | --- | --- | --- | --- | --- | --- | --- |
| **Women**  **n=32** | 1268 | 94 | 86 | 94 | 86 | 0.96  (0.89-1.0) | 0.0004 |
| **Men**  **n=46** | 2388 | 91 | 100 | 100 | 86 | 0.94  (0.86-1.0) | <0.0001 |

**Suppl. Fig. 1** ROC curves for the detection of severe aortic stenosis by ctCS in women and men.

**ctCS ≥1600 AU**  **ctCS ≥3000 AU** Men


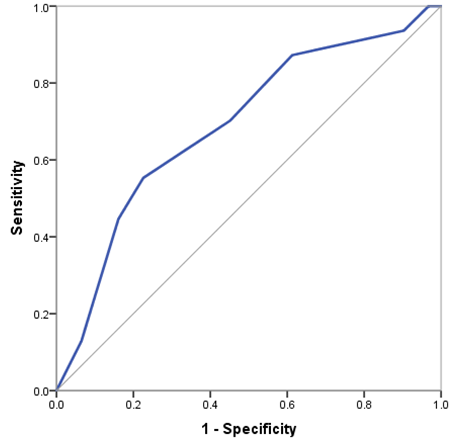

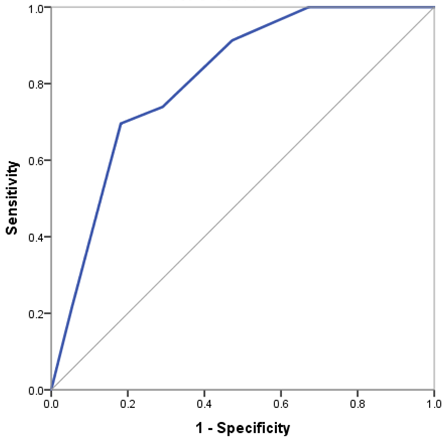


| **Threshold ctCS** | **Visual score** | | | | |
| --- | --- | --- | --- | --- | --- |
|  | **VS cut-off** | **Sensitivity, %** | **Specificity, %** | **AUC (95% CI)** | **p-value** |
| ≥1600 AU | 9 | 55 | 77 | 0.69 (0.57-0.81) | 0.005 |
| ≥3000 AU | 10 | 70 | 82 | 0.81 (0.72-0.9) | <0.0001 |

**Suppl. Fig. 2** ROC curves for the detection of ctCS thresholds by visual score.

**
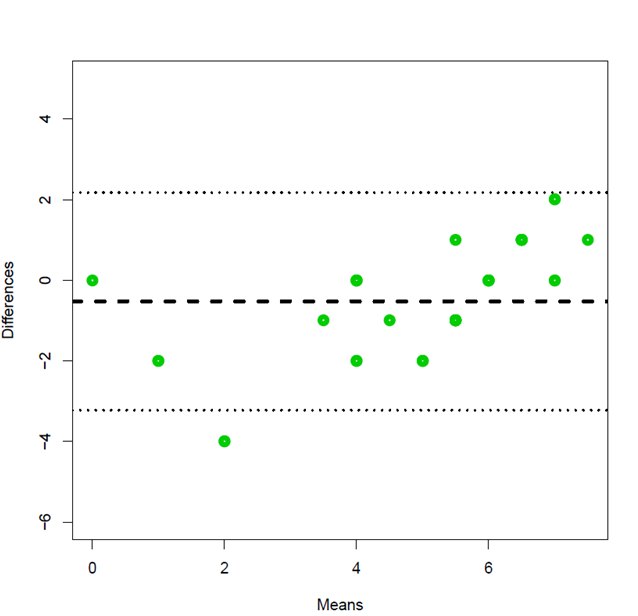
**

**Suppl. Fig. 3** Bland-Altman plot for inter-observer agreement in AV visual score without mobility pattern. 95% limits of agreement -3.22, 2.15, mean difference -0.53.
